# Diabetes and endometrial cancer: effect modification by body weight, physical activity and hypertension

**DOI:** 10.1038/sj.bjc.6603933

**Published:** 2007-10-02

**Authors:** E Lucenteforte, C Bosetti, R Talamini, M Montella, A Zucchetto, C Pelucchi, S Franceschi, E Negri, F Levi, C La Vecchia

**Affiliations:** 1 Istituto di Ricerche Farmacologiche ‘Mario Negri’, Milan, Italy; 2 Unità di Epidemiologia e Biostatistica, Centro di Riferimento Oncologico, Aviano (PN), Italy; 3 Unità di Epidemiologia, Istituto Nazionale Tumori ‘Fondazione Giovanni Pascale’, Naples, Italy; 4 International Agency for Research on Cancer, Lyon Cedex, France; 5 Unité d'épidémiologie du cancer, Institut de Médicine sociale et préventive (IUMSP), Université de Lausanne, Switzerland; 6 Istituto di Statistica Medica e Biometria ‘G. A. Maccacaro’, Università degli Studi di Milano, Milan, Italy

**Keywords:** case–control study, endometrial cancer, diabetes, body mass index, physical activity, hypertension

## Abstract

Among 777 endometrial cancer cases and 1550 controls from Italy and Switzerland, odds ratio was 1.7 (95% confidence interval: 1.2–2.5) for diabetes, and 5.1 for obese diabetic women as compared with non-obese non-diabetic ones. Diabetes shows a supramultiplicative effect with body mass index, but not with physical activity or hypertension.

An increased risk of endometrial cancer in diabetic women has often been reported (Parazzini *et al*, 1991, 1999; Brinton *et al*, 1992; La Vecchia *et al*, 1994; Shoff and Newcomb, 1998; Salazar-Martinez *et al*, 2000; Weiderpass *et al*, 2000; Anderson *et al*, 2001; Sharma *et al*, 2001; Cook *et al*, 2006; Friberg *et al*, 2007). Overweight and obesity are major risk factors for both conditions since they are related to increased endogenous oestrogen levels (Parazzini *et al*, 1991; Calle *et al*, 2003; Cook *et al*, 2006). Although the association between diabetes and endometrial cancer may be partly or largely accounted for by the higher body weight of endometrial cancer cases, it has been found to persist after adjustment for body mass index (BMI) (Brinton *et al*, 1992; La Vecchia *et al*, 1994; Parazzini *et al*, 1999; Salazar-Martinez *et al*, 2000; Friberg *et al*, 2007).

A few of the above studies have investigated the combined role of diabetes and BMI on risk, finding a higher risk among obese diabetic women, most of these, but not all (Anderson *et al*, 2001), reporting some excess risk also among non-overweight diabetic women.

Diabetes is associated with hypertension and physical inactivity, which in turn have been related to endometrial cancer risk (Parazzini *et al*, 1999; Cust *et al*, 2007; Voskuil *et al*, 2007). In a Swedish cohort study of 225 cases of endometrial cancer, the relative risk was 2.7 for diabetic women reporting low physical activity and 1.1 among those reporting high physical activity (Friberg *et al*, 2007). In an Italian case–control study, the association with diabetes was of similar magnitude in women with or without hypertension (Parazzini *et al*, 1999).

We therefore investigated whether the association between diabetes and endometrial cancer was modified by BMI, physical activity and hypertension in two case–control studies from Italy and Switzerland.

## MATERIALS AND METHODS

We analysed the combined data of two case–control studies of endometrial cancer, the first conducted in the greater Milan area and the Swiss Canton of Vaud between 1988 and 1994 on 410 cases and 753 controls (Augustin *et al*, 2003), the second conducted in the provinces of Pordenone and Milan, in northern Italy, and Naples, in southern Italy, in 1992–2006 on 454 cases and 908 controls. A subset from the latter included in a previous study was excluded (Dal Maso *et al*, 2004). This left a total of 777 women (aged 18–79 years, median 61 years) with histologically confirmed endometrial cancer, with no previous diagnosis of cancer, and 1550 control women (aged 18–80 years, median 61 years) admitted to the same network of hospitals as cases for acute, non-neoplastic, and non-gynaecologic, non-hormone-related, non-metabolic conditions. Women with a history of hysterectomy were excluded from the control group. Fewer than 5% of subjects approached for interview refused to participate.

For both cases and controls, data were collected by trained interviewers during their hospital stay using similar structured questionnaires covering education, socio-economic factors, lifestyle habits, anthropometric measures and physical activity at various ages, a validated food-frequency section, menstrual and reproductive history, and use of oral contraceptive (OC) and hormone replacement therapy (HRT). Details were also collected on personal medical history, including diabetes and hypertension. Body mass index at diagnosis and at age 30–39 years were computed as weight/height^2^ (kg m^−2^); physical activity at age 30–39 years was considered.

Odds ratios (OR) according to history of diabetes and corresponding 95% confidence intervals (CIs), were estimated using multiple logistic regression models. The models included terms for age, year of interview, study centre, years of education, parity, menopausal status, OC and HRT use, plus BMI, physical activity and history of hypertension when appropriate. To test for heterogeneity, we compared the differences between the −2 log likelihood of the models with and without interaction term with the *χ*^2^ distribution with one degree of freedom.

## RESULTS

Table 1 shows the distribution of 777 endometrial cancer cases and 1550 controls according to age, history of diabetes, BMI at diagnosis, BMI at age 30–39 years, physical activity at age 30–39 years and history of hypertension, and the corresponding ORs. By design, cases and controls had similar age distribution. Cases reported more frequently than controls a history of diabetes (9.9 *vs* 4.6%), with an OR of 1.7 (95% CI: 1.2–2.5) after detailed adjustment for BMI at diagnosis. The OR for diabetes was 2.0 (95% CI: 1.4–2.9) in the absence of adjustment for BMI, and 1.9 (95% CI: 1.3–2.7) when a term for BMI at age 30–39 was included in the model. The OR was 2.4 (95% CI: 1.9–3.1) for women with a BMI at diagnosis ⩾30 kg m^−2^, 1.6 (95% CI: 1.3–2.0) for women with a BMI at age 30–39 ⩾25 kg m^−2^, 1.4 (95% CI: 1.1–1.8) for women with a low level of physical activity at age 30–39, and 1.2 (95% CI: 1.0–1.5) for women with a history of hypertension.

Table 2 considers the combined effect of diabetes with BMI at diagnosis, BMI at age 30–39, physical activity at age 30–39, and history of hypertension. Compared with non-diabetic non-obese women (BMI at diagnosis <30 kg m^−2^), the OR was 1.4 for non-obese women with diabetes, but rose to 5.1 for obese diabetic women. Corresponding ORs for women with diabetes were 1.8 with BMI at age 30–39 years <25 kgm^−2^, and 3.4 with BMI at age 30–39 years ⩾25 kg m^−2^. However, the interaction between diabetes and BMI at diagnosis or at 30–39 years was not statistically significant. As compared with non-diabetic women with moderate or high physical activity, the OR was similar in diabetic women reporting moderate/high (OR=1.9) or low physical activity (OR=2.0). Similarly, the ORs were comparable in diabetic women without (OR=1.8) and with (OR=2.1) a history of hypertension, as compared with non-diabetic women with no hypertension.

The relation between diabetes and endometrial cancer risk was also examined in strata of age, menopausal status, parity, OC and HRT use, but no meaningful differences in risk emerged.

## DISCUSSION

This large case–control study on endometrial cancer confirms and provides further quantitative evidence that diabetes is independently related to endometrial cancer risk. The association was partly, but not totally, explained by overweight. As in other epidemiological studies (Shoff and Newcomb, 1998; Salazar-Martinez *et al*, 2000; Anderson *et al*, 2001; Friberg *et al*, 2007), the association with diabetes was stronger for obese women, suggesting a supramultiplicative effect between diabetes and obesity in endometrial cancer risk.

Of particular interest, our information on BMI at both diagnosis and in the distant past allowed us to show a significant association between diabetes and endometrial cancer for women who were not overweight at age 30–39. The combination of diabetes and overweight in young/middle age is compatible with a multiplicative model for exposure to both factors on the relative risk of endometrial cancer. However, these subgroup analyses were affected by large random variation, so we could not distinguish between different models.

Type II diabetes is related to hyperinsulinaemia, which may increase free oestrogen levels by decreasing the concentration of sex hormone-binding globulin (Nestler *et al*, 1991; Friberg *et al*, 2007). Hyperinsulinaemia may also influence the insulin –growth factor (IGF) system. Increasing levels of IGF-1 (Weiderpass *et al*, 2003) and of IGF-binding protein-1 (Augustin *et al*, 2004) have been associated with endometrial cancer risk, particularly in older overweight women. Overweight and obesity have also been related to low levels of the insulin sensitiser adiponectin, and consequently to increased risk of hyperinsulinaemia and type II diabetes (Mantzoros *et al*, 2005). An inverse association has also been shown between adiponectin levels and endometrial cancer risk (Dal Maso *et al*, 2004). These mechanisms do not only explain the association between diabetes and endometrial cancer, but also provide a background for a positive interaction with overweight.

The Swedish Mammography Cohort Study (Friberg *et al*, 2007) found a strongly positive interaction between diabetes and low physical activity, and endometrial cancer risk, the relative risk for obese diabetics report low physical activity being 9.6. In our study, however, the association with diabetes was not modified by physical activity. This apparent difference may be due to different measures of the physical activity level in various populations, and also to the play of chance, because the two strata of physical activity in the Swedish study included 5 and 17 diabetic cases only. Likewise, with reference to hypertension, we were able to confirm the absence of a meaningful interaction with diabetes (Parazzini *et al*, 1999).

This is a hospital-based case–control study with some of the related weaknesses and strengths. Diabetics may be more frequently admitted to hospital, although we excluded from the comparison group all women hospitalised for chronic and metabolic conditions. Furthermore, the 4.6% prevalence of diabetes in middle age and elderly women in our comparison group is similar to that of population-based surveys (La Vecchia *et al*, 1995). The same interview setting allowed collection of comparable medical history details, which were shown to be satisfactorily reproducible for diabetes (*k*=0.85) (Bosetti *et al*, 2001). Furthermore, cases and controls came from similar catchment areas, and participation rate was almost complete (approximately 95% for cases and controls). We were also able to allow for confounding by major hormonal and reproductive factors for endometrial cancer, and for BMI, not only at diagnosis but also in the past.

## Figures and Tables

**Table 1:** Distribution of 777 cases of endometrial cancer and 1550 controls according to age, history of diabetes and other selected variables. Italy and Switzerland, 1988–2006

**Characteristic**	**Case number (%)**	**Control number (%)**	**OR^a^ (95% CI)**
*Age (years)*			
<40	24 (3.1)	73 (4.7)	
40–49	75 (9.6)	174 (11.2)	
50–59	233 (30.0)	444 (28.6)	
60–69	312 (40.1)	589 (38.0)	
⩾70	133 (17.1)	270 (17.4)	
			
*History of diabetes*			
No	700 (90.1)	1479 (95.4)	1^b^
Yes	77 (9.9)	71 (4.6)	1.7 (1.2–2.5)
			
*BMI at diagnosis (kg m*^−*2*^)^c^			
< 30	555 (71.8)	1325 (85.89)	1^b^
⩾30	218 (28.2)	222 (14.2)	2.4 (1.9–3.1)
			
*BMI (kg m*^−*2*^) *at age 30–39*^c^			
< 25	532 (71.2)	1152 (79.6)	1^b^
⩾25	215 (28.8)	295 (10.4)	1.6 (1.3–2.0)
			
*Physical activity at age 30–39* c			
Moderate/high	543 (70.2)	1219 (79.2)	1^b^
Low	230 (29.7)	321 (20.8)	1.4 (1.1–1.8)
			
*History of hypertension*			
No	519 (66.8)	1169 (75.4)	1^b^
Yes	258 (33.2)	381 (24.6)	1.2 (1.0–1.5)

BMI, body mass index; CI, confidence interval; HRT, hormone replacement therapy; OC, oral contraceptive; OR, odds ratio.

a Estimates from logistic regression models, including terms for the above factors plus year of interview, study centre, education, parity, menopausal status, OC and HRT use.

b Reference category.

c The sum does not add up to the total because of some missing values.

**Table 2:** ORs and corresponding 95% CIs among 777 cases of endometrial cancer and 1550 controls according to the combined effect of history of diabetes, BMI, physical activity or history of hypertension. Italy and Switzerland, 1988–2006

	**No diabetes**			**Diabetes**		
	**Cases**	**Controls**	**OR^a^ (95% CI)**	**Cases**	**Controls**	**OR^a^ (95% CI)**
*BMI at diagnosis (kg m*^−*2*^)^b^						
<30	526	1278	1^c^	29	47	1.4 (0.9–2.4)
⩾30	170	196	2.3 (1.8–3.0)	48	24	5.1 (3.0–8.7)
						
*BMI (kg m*^−*2*^) *at age 30–39*^b^						
<25	498	1115	1^c^	34	37	1.8 (1.1–3.0)
⩾25	176	269	1.6 (1.3–2.0)	39	26	3.4 (2.0–5.8)
						
*Physical activity at age 30–39* b						
Moderate/high	491	1169	1^c^	52	50	1.9 (1.3–3.0)
Low	205	300	1.5 (1.2–1.8)	25	21	2.0 (1.0–3.8)
						
*History of hypertension*						
No	485	1134	1^c^	34	35	1.8 (1.1–3.1)
Yes	215	345	1.3 (1.0–1.6)	43	36	2.1 (1.3–3.5)

BMI, body mass index; CI, confidence interval; HRT, hormone replacement therapy; OC, oral contraceptive; OR, odds ratio.

a Estimates from logistic regression models, adjusted by age, year of interview, study centre, education, parity, menopausal status, OC and HRT use, plus current and past BMI, physical activity and history of hypertension when appropriate.

b The sum does not add up to the total because of some missing values.

c Reference category.

## References

[bib1] Anderson KE, Anderson E, Mink PJ, Hong CP, Kushi LH, Sellers TA, Lazovich D, Folsom AR (2001) Diabetes and endometrial cancer in the Iowa women's health study. Cancer Epidemiol Biomarkers Prev 10: 611–616 11401910

[bib2] Augustin LS, Dal Maso L, Franceschi S, Talamini R, Kendall CW, Jenkins DJ, Vidgen E, La Vecchia C (2004) Association between components of the insulin-like growth factor system and endometrial cancer risk. Oncology 67: 54–59 15459496 10.1159/000080286

[bib3] Augustin LS, Gallus S, Bosetti C, Levi F, Negri E, Franceschi S, Dal Maso L, Jenkins DJ, Kendall CW, La Vecchia C (2003) Glycemic index and glycemic load in endometrial cancer. Int J Cancer 105: 404–407 12704677 10.1002/ijc.11089

[bib4] Bosetti C, Tavani A, Negri E, Trichopoulos D, La Vecchia C (2001) Reliability of data on medical conditions, menstrual and reproductive history provided by hospital controls. J Clin Epidemiol 54: 902–906 11520649 10.1016/s0895-4356(01)00362-6

[bib5] Brinton LA, Berman ML, Mortel R, Twiggs LB, Barrett RJ, Wilbanks GD, Lannom L, Hoover RN (1992) Reproductive, menstrual, and medical risk factors for endometrial cancer: results from a case-control study. Am J Obstet Gynecol 167: 1317–1325 1442985 10.1016/s0002-9378(11)91709-8

[bib6] Calle C, Campion J, Garcia-Arencibia M, Maestro B, Davila N (2003) Transcriptional inhibition of the human insulin receptor gene by aldosterone. J Steroid Biochem Mol Biol 84: 543–553 12767279 10.1016/s0960-0760(03)00072-4

[bib7] Cook LS, Weiss NS, Doherty JA, Chen C (2006) Endometrial cancer. In: Schottenfeld D, Fraumeni Jr JF (eds). Cancer epidemiology and prevention, pp 1027–1043. New York: Oxford University Press

[bib8] Cust AE, Armstrong BK, Friedenreich CM, Slimani N, Bauman A (2007) Physical activity and endometrial cancer risk: a review of the current evidence, biologic mechanisms and the quality of physical activity assessment methods. Cancer Causes Control 18: 243–258 17206535 10.1007/s10552-006-0094-7

[bib9] Dal Maso L, Augustin LS, Karalis A, Talamini R, Franceschi S, Trichopoulos D, Mantzoros CS, La Vecchia C (2004) Circulating adiponectin and endometrial cancer risk. J Clin Endocrinol Metab 89: 1160–1163 15001602 10.1210/jc.2003-031716

[bib10] Friberg E, Mantzoros CS, Wolk A (2007) Diabetes and risk of endometrial cancer: a population-based prospective cohort study. Cancer Epidemiol Biomarkers Prev 16: 276–280 17301260 10.1158/1055-9965.EPI-06-0751

[bib11] La Vecchia C, Decarli A, Franceschi S, Ferraroni M, Pagano R (1995) Prevalence of chronic diseases in alcohol abstainers. Epidemiology 6: 436–438 7548357 10.1097/00001648-199507000-00021

[bib12] La Vecchia C, Negri E, Franceschi S, D'Avanzo B, Boyle P (1994) A case-control study of diabetes mellitus and cancer risk. Br J Cancer 70: 950–953 7947103 10.1038/bjc.1994.427 PMC2033532

[bib13] Mantzoros CS, Li T, Manson JE, Meigs JB, Hu FB (2005) Circulating adiponectin levels are associated with better glycemic control, more favorable lipid profile, and reduced inflammation in women with type 2 diabetes. J Clin Endocrinol Metab 90: 4542–4548 15914524 10.1210/jc.2005-0372

[bib14] Nestler JE, Powers LP, Matt DW, Steingold KA, Plymate SR, Rittmaster RS, Clore JN, Blackard WG (1991) A direct effect of hyperinsulinemia on serum sex hormone-binding globulin levels in obese women with the polycystic ovary syndrome. J Clin Endocrinol Metab 72: 83–89 1898744 10.1210/jcem-72-1-83

[bib15] Parazzini F, La Vecchia C, Bocciolone L, Franceschi S (1991) The epidemiology of endometrial cancer. Gynecol Oncol 41: 1–16 2026352 10.1016/0090-8258(91)90246-2

[bib16] Parazzini F, La Vecchia C, Negri E, Riboldi GL, Surace M, Benzi G, Maina A, Chiaffarino F (1999) Diabetes and endometrial cancer: an Italian case-control study. Int J Cancer 81: 539–542 10225441 10.1002/(sici)1097-0215(19990517)81:4<539::aid-ijc6>3.0.co;2-q

[bib17] Salazar-Martinez E, Lazcano-Ponce EC, Lira-Lira GG, Escudero-De los Rios P, Salmeron-Castro J, Larrea F, Hernandez-Avila M (2000) Case-control study of diabetes, obesity, physical activity and risk of endometrial cancer among Mexican women. Cancer Causes Control 11: 707–711 11065007 10.1023/a:1008913619107

[bib18] Sharma DN, Chander S, Gairola M, Kumar L, Parida DK, Pathy S (2001) Medical disorders associated with endometrial carcinoma. J Assoc Physicians India 49: 630–633 11584939

[bib19] Shoff SM, Newcomb PA (1998) Diabetes, body size, and risk of endometrial cancer. Am J Epidemiol 148: 234–240 9690359 10.1093/oxfordjournals.aje.a009630

[bib20] Voskuil DW, Monninkhof EM, Elias SG, Vlems FA, van Leeuwen FE (2007) Physical activity and endometrial cancer risk, a systematic review of current evidence. Cancer Epidemiol Biomarkers Prev 16: 639–648 17416752 10.1158/1055-9965.EPI-06-0742

[bib21] Weiderpass E, Brismar K, Bellocco R, Vainio H, Kaaks R (2003) Serum levels of insulin-like growth factor-I, IGF-binding protein 1 and 3, and insulin and endometrial cancer risk. Br J Cancer 89: 1697–1704 14583772 10.1038/sj.bjc.6601312 PMC2394399

[bib22] Weiderpass E, Persson I, Adami HO, Magnusson C, Lindgren A, Baron JA (2000) Body size in different periods of life, diabetes mellitus, hypertension, and risk of postmenopausal endometrial cancer (Sweden). Cancer Causes Control 11: 185–192 10710204 10.1023/a:1008946825313

